# Training in endoscopic mucosal resection: effectiveness and clinical utility of a short course for practicing endoscopists

**DOI:** 10.1093/jcag/gwaf015

**Published:** 2025-06-21

**Authors:** Ahmed Kayal, Sylvain Coderre, Maitreyi Raman, Heather L Hill, Stephanie Jaunin, Diana Kerrison, Adrian Harvey, Kevin McLaughlin, Steven J Heitman

**Affiliations:** Department of Medicine, Division of Gastroenterology and Hepatology, University of Calgary, Calgary, Alberta T2N 1N4, Canada; Department of Community Health Sciences, University of Calgary, Calgary, Alberta T2N 4Z6, Canada; Department of Medicine, Rabigh Branch, King Abdulaziz University, Jeddah 21589, Saudi Arabia; Department of Medicine, Division of Gastroenterology and Hepatology, University of Calgary, Calgary, Alberta T2N 1N4, Canada; Department of Community Health Sciences, University of Calgary, Calgary, Alberta T2N 4Z6, Canada; Department of Medicine, Division of Gastroenterology and Hepatology, University of Calgary, Calgary, Alberta T2N 1N4, Canada; Department of Community Health Sciences, University of Calgary, Calgary, Alberta T2N 4Z6, Canada; Advanced Technical Skills Simulation Laboratory, Cumming School of Medicine, University of Calgary, Calgary, Alberta T2N 4N1, Canada; Advanced Technical Skills Simulation Laboratory, Cumming School of Medicine, University of Calgary, Calgary, Alberta T2N 4N1, Canada; Forzani & MacPhail Colon Cancer Screening Centre, Alberta Health Services (Calgary Zone), Calgary, Alberta T2N 5A1, Canada; Department of Surgery, University of Calgary, Calgary, Alberta T2N 2T9, Canada; Department of Oncology, University of Calgary, Calgary, Alberta T2N 4N1, Canada; Department of Community Health Sciences, University of Calgary, Calgary, Alberta T2N 4Z6, Canada; Department of Medicine, Division of Nephrology, University of Calgary, Calgary, Alberta T2N 2T8, Canada; Department of Medicine, Division of Gastroenterology and Hepatology, University of Calgary, Calgary, Alberta T2N 1N4, Canada; Department of Community Health Sciences, University of Calgary, Calgary, Alberta T2N 4Z6, Canada; Forzani & MacPhail Colon Cancer Screening Centre, Alberta Health Services (Calgary Zone), Calgary, Alberta T2N 5A1, Canada

**Keywords:** colorectal, endoscopic mucosal resection, curriculum, training

## Abstract

**Background and Aims:**

Endoscopic mucosal resection (EMR) is not systematically taught during most training programs. The aim of this study was to evaluate the effectiveness and clinical utility of a 1-day didactic and simulation-based EMR curriculum for practicing endoscopists without prior formal training in advanced endoscopic tissue resection.

**Methods:**

We designed a 1-day lecture and simulation-based EMR course. Twelve participants completed the course. Effectiveness and clinical utility were evaluated using sequential explanatory mixed methods. All participants completed a pre-course multiple choice question (MCQ) examination followed by a separate, post-course MCQ examination with a similar blueprint. A survey was also conducted to assess cognitive fatigue, perceived benefit, and potential for change in EMR practice. Finally, a delayed MCQ examination was administered 10-14 weeks later to assess knowledge retention and qualitative data were sequentially collected from 3 candidates via semi-structured interviews.

**Results:**

The mean pre-course score was 47.8% (SD 12.4%). The mean post-course score was 75% (9.9%) and the mean delayed score was 70.8% (13.6%), both significantly higher than the mean pre-course score (*P* < .001; Cohen’s *d* = 1.86 and *P* < .001; Cohen’s *d* = 1.47, respectively). There was no significant difference between the mean post- and delayed-course test scores (*P* = .2). Three themes emerged from the interviews: (1) a need for EMR training, (2) improved knowledge evaluating polyps, and (3) changed or refined EMR technique after the course.

**Conclusions:**

This study demonstrates significant knowledge acquisition and retention of cognitive skills and suggests a change in practice following a 1-day focused didactic and simulation-based EMR course.

## Introduction

Endoscopic mucosal resection (EMR) is an important technique for the removal of pre-malignant polyps during colonoscopy. Although piecemeal EMR of large (≥ 20 mm) non-pedunculated colorectal polyps (LNPCPs) is considered an advanced skill, the principles of EMR are relevant to any polypectomy involving a submucosal lift followed by hot snare resection^.1,2^ Basic polypectomy skills are a core competency in all gastroenterology and many general surgery training programs, but EMR has not been a focus historically.^3^ Variable exposure to more complex polypectomy including piecemeal EMR of LNPCPs may occur when additional training is pursued during advanced endoscopy fellowships, but programs traditionally emphasize pancreaticobiliary endoscopy including endoscopic retrograde cholangiopancreatography and endoscopic ultrasound. Nevertheless, graduates of these programs are often presumed to be competent in complex polypectomy, even when some may have only completed a handful of cases, with no structured training prior to independent practice.

It is now firmly established that the overwhelming majority of all LNPCPs, irrespective of size, can and should be managed endoscopically given similar effectiveness, lower risks, and substantially reduced costs compared to surgery.^4–6^ Moreover, with many colorectal cancer (CRC) screening programs utilizing stool-based tests which when positive increase the burden of polyps including LNPCPs during colonoscopy,^7^ it is essential that all endoscopists performing screening-related colonoscopy understand the role of interventional endoscopy in treating these patients. Although a small subset of complex LNPCPs is more appropriate for highly experienced experts, most lesions encountered can be treated by appropriately trained generalists who possess refined cognitive and technical skills. There is a growing need for upskilling in EMR, in an era where high colonoscopy quality and optimal outcomes are expected.

Endoscopic training often occurs in the procedural suite with skills acquired experientially under the supervision of a more senior mentor. This apprenticeship model is impractical for most endoscopists already in independent practice. In contrast, teaching through succinct lectures and instructional videos supplemented by hands-on training in a simulation environment allows for targeted and efficient learning of technical skills while avoiding the risks to patients inherent during the early learning curve.^8,9^ Short courses in basic polypectomy and EMR are currently offered by several professional societies: eg, Canadian Association of Gastroenterology (CAG)—Endoscopic Polypectomy Improvement Course; American Society of Gastrointestinal Endoscopy (ASGE)—Skills, Training, Assessment and Reinforcement (STAR), Lower GI EMR STAR. However, despite the popularity of these courses empirical evidence supporting their effectiveness is lacking. In addition, it is unclear if appropriate educational psychology frameworks or education theories were utilized in designing these courses.

The purpose of this study was to develop and evaluate the effectiveness and clinical utility of a 1-day combined multi-modal didactic and ex-vivo simulation course in piecemeal EMR for practicing endoscopists. We compared the cognitive knowledge base before and after the course, assessed long-term retention of knowledge and obtained a qualitative assessment of the course’s impact on the clinical practice of participants.

## Methods

### Overview of colorectal EMR course

The course took place at the Forzani & MacPhail Colon Cancer Screening Centre (CCSC) and in the Advanced Technical Skills Simulation Laboratory (ATSSL) at the University of Calgary. A planning committee (A.K., S.C., and S.J.H.) was formed to develop the course. The committee assessed information from: (1) a review of the relevant literature; (2) a needs assessment survey which was circulated to all gastroenterologists and colorectal surgeons who perform screening-related colonoscopy in Calgary (Supplementary Material S1); and (3) their expertise in the field. The course curriculum was developed according to Kern’s 6-step framework.^10^ Key topics were identified in parallel leading to the creation of a series of lectures and instructive videos. Simultaneously, a high-fidelity ex-vivo porcine model comprised of distal sigmoid colon and rectum was designed and constructed by ATSSL staff members (H.H., S.J.) in collaboration with an experienced endoscopy nurse (D.K.) and clinical experts in EMR (A.K., S.J.H.) (Supplementary Material S2). Prior to offering the course as part of the study, a logistical pilot run was undertaken to enhance consistency, confirm timing, and troubleshoot the model.

The 1-day course was offered free of charge and was accredited for Continuing Medical Education hours by the University of Calgary (Supplemental Material S3). An invitation to participate was circulated via email to all endoscopists in Calgary. Trainees, therapeutic endoscopists, and those who had taken prior short courses in EMR were excluded. The course encompassed a full-day running from 07:30 to 16:00. Lectures and short video presentations were supplemented by interactive discussion periods in the morning. This was followed by hands-on training in the ATSSL in the afternoon supervised by mentors experienced in EMR. Participants provided informed consent to have their data collected and the study was approved by the Health Research Ethics Board of Alberta (HREBA.CC-19-0511).

### Study design and data collection

**Figure 1.**
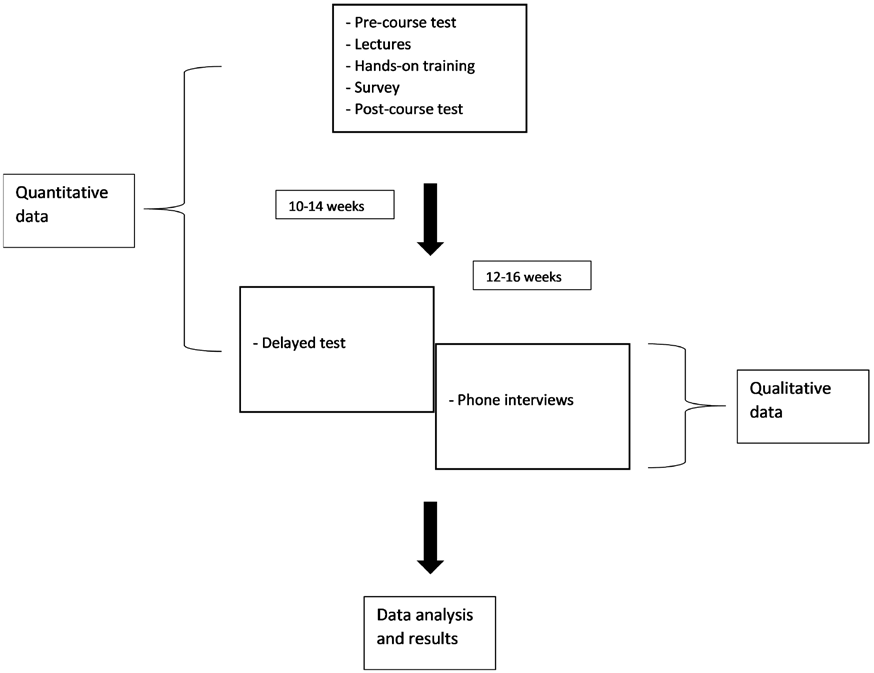
Roadmap for the University of Calgary EMR course.

This was a prospective sequential explanatory mixed methods study (QUAN: qual) (Figure 1). For the first quantitative phase, a 26 multiple choice question (MCQ) examination with 4 options and 1 key answer was given to all participants at the start of the course to establish pre-course base knowledge. Second, a parallel form MCQ examination using the same blueprint was administered at the end of the course. This assessed post-course knowledge acquisition. Lastly, a delayed-course examination was completed 10-14 weeks after the course to evaluate knowledge retention. The delayed examination was the same as the pre-course examination, a method previously used^11^ and justified given the time interval and content introduced between the 2 administrations of the test. The standards of the MCQ examinations were set using the modified Nedelsky’s method,^12^ which allowed consistency of item and overall examination difficulty for both parallel examinations. All items were developed as per the National Board of Medical Examiners’s recommendations and rules.^13^

Three members of the authors (S.H., A.K., and S.C.) developed the MCQs and then evaluated the tests with Nedlesky’s method. S.H. and A.K. are content experts and S.C. is a medical education expert. An agreement was reached on the minimal competent candidate being a general gastroenterologist with no additional training or regular exposure to large colorectal lesions or EMR (S.C. met this criterion). In addition, the variance in experience between candidates was considered in establishing the borderline candidate. After mean performance levels (MPL) were evaluated by the judges, changes to few items were made after discussion and an overall MPL for the 2 different tests was similar and parallel forms of examinations were established (Supplementary Material S6).

At the completion of the course, a survey was administered to quantitatively assess the educational benefits and potential change or refinement of participants’ approach to the management of LNPCPs. A 5-point Likert scale system was used: 5 (strongly agree) to 1 (strongly disagree). In addition, cognitive fatigue was assessed using a validated 8-point Likert scale,^14^ where 7 means extremely tired and 0 means not tired at all (Supplementary Material S4).

Lastly, a second qualitative phase was conducted 12-16 weeks after the course in order to: (1) enrich and triangulate the quantitative findings; and (2) assess the satisfaction and utility of the newly acquired EMR knowledge and skills. The number of interviews conducted was determined by the principle of data saturation.^15^ We pursued a “code saturation” rather than a “meaning saturation” approach. This was influenced by the number of candidates, iterative sampling (gender and years of experience were considered), researcher-driven deductive inquiry, and the identification of concrete codes.^16^

### Statistical and qualitative analysis

The primary outcome was the change in MCQ scores between the post- and pre-course examinations. Estimating a mean baseline MCQ score of 40%, a mean post-course test score of 60% and an SD of 15% for each exam, we determined that a sample size of 10 would have 96% power to detect a significant difference in mean MCQ scores associated with participation in the course.

Descriptive statistics were used to describe the demographics of the participants. Comparison between the mean scores of the MCQ examinations was performed by paired *t*-test. A *P* value of < .05 was considered significant. Data analysis was performed using STATA version 14.0 (StataCorp LP, College Station, TX).

For the qualitative data, the phone interviews were transcribed as clean verbatim during the encounter. We performed coding and thematic analysis of narrative questionnaire data and the transcripts of the phone interviews^17^ and used a constant comparative analytical approach to identify the emerging themes. Two investigators (A.K., K.M.) co-coded the data independently before reaching a consensus on the main thematic elements.

### Credibility in qualitative analysis

In qualitative research, the conceptual framework is constructed through the researchers’ interpretation of data, leading to unavoidable subjectivity. To address this and limit bias, different strategies were implemented.^18^ First, reflexivity statements were reported by the researchers to reflect on their backgrounds and help readers review the results in a consistent context (Supplementary Material S5). Second, co-coding of the data was done independently. Third, triangulation of qualitative findings with the quantitative data from the exit survey enhanced credibility (Table 3). Finally, member checking was done where the preliminary data were evaluated by the participants, and all agreed the emerged themes captured their perspective of the study.

## Results

### Participants

Of the email invitations sent to endoscopists across Calgary, 16 responded expressing interest and the first 12 were invited to participate. We selected 12 instead of 10 to account for potential dropouts. The study cohort was then divided into 2 equal groups of 6 participants. Seven out of 12 self-identified as women (58.3%). All participants were practicing gastroenterologists with no prior training or courses in EMR but with variable years of general endoscopy/clinical experience (Table 1).

**Table 1.** Demographics and participants’ characteristics.

	Frequency (%)
**Demographics**	
** Male**	5 (41.67%)
** Female**	7 (58.33%)
**Endoscopy experience**	
** Less than 2 years**	2 (16.67%)
** 2–5 years**	2 (16.67%)
** 6–10 years**	3 (25%)
** 11–15 years**	2 (16.67%)
** Over 15 years**	3 (25%)
**Specialty**	
** Gastroenterology**	12 (100%)
** Surgery**	0 (0%)
**Performance of EMR of 2 cm polyps or larger per month (before the course)**	
** 0**	2 (16.67%)
** 1–2**	5 (41.67%)
** 3–5**	5 (41.67%)
** More than 5**	0 (0%)

### Quantitative phase

**Figure 2.**
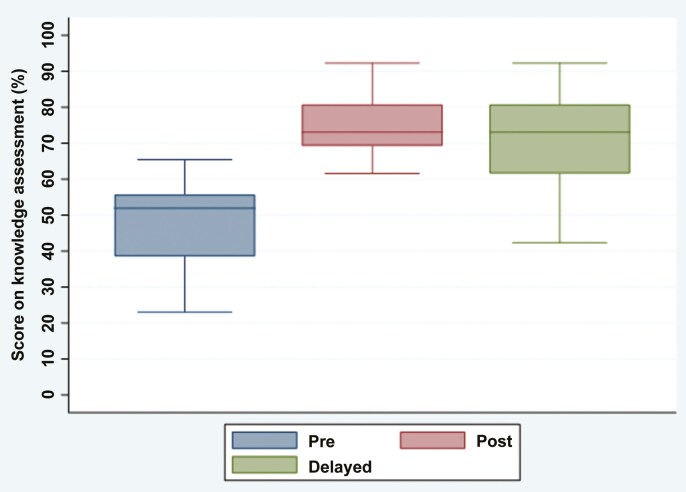
Pre, post, and delayed test scores (mean ± SD).

The mean pre-course test score was 47.76% (SD 12.43%). The mean post-course test score was 75% (9.91%) and the mean delayed test score was 70.83% (13.57%). There was a statistically significant increase in the mean scores between pre- and post-course tests (*P* < .001, Cohen’s *d* = 1.86), and between pre-course and delayed tests (*P* < .001, Cohen’s *d* = 1.47). There was no significant difference between post-course and delayed-course test mean scores (*P* = .2) (Figure 2, Table 2). Please see Table 3 for a summary of the post-course survey findings.

**Table 2.** Pre, post, and delayed test scores.

	Pre	Post	Delayed
1	15	19	22
2	17	24	24
3	10	16	11
4	14	18	18
5	14	19	15
6	11	19	16
7	6	20	21
8	16	22	19
9	14	17	19
10	10	18	16
11	13	18	19
12	9	24	21
Mean % (SD)	47.76% (12.4%)	75% (9.91%)	70.83% (13.57%)

**Table 3.** Post-course survey summary^*^.

Survey topic	Mean (SD)	Supporting qualitative data
Cognitive fatigue^**^	3.55 (1.51)	“For me it was ok, it was appropriately targeted and the key thing that all the candidates were experienced GIs. I do not think fellows would have handled it as well”
The program met the objectives	4.92 (0.29)	“I thought the content was excellent”
Useful in terms of knowledge	5 (0)	“Yes absolutely, I think more of different classifications now”
Useful in terms of technical skills	4.83 (0.39)	“Yes, this is good for small polyps too”
Material and topics were clear	4.92 (0.29)	“I feel you did a good job there”
Change/refine EMR technique after the course	4.75 (0.45)	“Now I look at polyps and think about different classifications. The course gave me a good approach”
The EMR model was similar to a real polyp^***^	4.18 (0.60)	“It was good but behaves a little different”
Prefer the whole course in 1 day	4.5 (0.90)	“I think one day is better”
Participation in future EMR courses	4.75 (0.45)	

^*^Likert scale scores out of 5. 5 is “strongly agree” and 1 is “strongly does not agree.”

^**^Cognitive fatigue score is out of 7.

^***^One participant did not attend the hands-on session. The mean score was adjusted accordingly.

### Qualitative phase

Participants were randomly selected and underwent a 15- to 20-minute phone interview between 12 and 16 weeks after the course. “Code saturation” was reached after 3 interviews. Three themes emerged after coding and thematic analysis of the transcribed encounters: (1) colorectal EMR is not taught formally during training, (2) the course resulted in increased knowledge and confidence in colorectal polyp evaluation and management, and (3) the course changed or refined the approach and technique of EMR.

#### Theme 1: No formal training

Participants consistently expressed that the material covered in the course was useful in terms of improving knowledge and skills that had not been previously taught in their training. It was cited that the material was advanced but appropriately targeted for practicing endoscopists.

“This course fits a niche that is not taught in any organized way.”

“There were a lot of different classifications and basic knowledge that needed to be reviewed. Like Kudo and Paris. The risk of cancer and the position of polyps and others were very good to know. That was not taught in our training.”

#### Theme 2: Gained knowledge and increased confidence

Participants stated that they are now more confident and take their time in evaluating and approaching all polyps in general and for larger polyps in particular; even for polyps that may be too difficult. The knowledge gained from the course enabled them to consider different treatment options more confidently, including attempting to resect if it can be done competently and safely, referring to another endoscopist, or even referring to surgery for lesions deemed not suitable for EMR. In addition, many expressed that the MCQ examination “made more sense” after the course.

“The course was a very good refresher and I have more confidence now.”

“I would say my ability to evaluate polyps and resection is much better. This reinforces that a systemic approach is important like position, resectability, or do we think it is cancer or not or whether should we be attempting to resect or not. Now I have a plan for most types of polyps rather than just refer them.”

#### Theme 3: Changed or refined approach and resection techniques

All participants expressed that their approach and resection technique did change. Although the course was designed for large polyps, participants felt that the resection techniques were applicable to smaller polyps as well and used the newly gained skills for polypectomy in general.

“Yes, I think this will serve as a much more systematic approach to polypectomy and enhance the skill set. It’s a great use on smaller polyps as well.”

## Discussion

Using best practices in education psychology we developed a 1-day curriculum for training in EMR and evaluated its effectiveness and clinical utility. Among this group comprised of practicing endoscopists without prior formal training in EMR, a substantial improvement in mean MCQ test scores was demonstrated. While perhaps expected, our immediate post-course MCQ scores were substantially higher than the baseline test results. In medical education, we strive for deeper learning reflected in the ability to retrieve and apply knowledge long after an educational intervention. Thus, it is encouraging that our 3- to 4-month-delayed examination results were also higher than the pre-course scores. This indicates that participants retained the knowledge delivered in our curriculum. Furthermore, our delayed qualitative interviews with participants point to practice change or refinement in the approach to managing LNPCPs.

Short courses in endoscopy are common, including those offered by subspecialty society associations (eg, ASGE, CAG) that focus on tissue resection. Our primary aim was not to develop another course, but rather to generate empirical data on the clinical effectiveness and utility of a short course in EMR. Until now, such data had been lacking. Therefore, in designing our curriculum and planning its delivery we were careful to adhere to key cognitive psychology principles and education theories.^19–22^ While a comprehensive discussion of these is beyond the scope of this manuscript, several elements deserve emphasis. Desy et al.^19^ present a framework for learning that comprises 2 major phases: knowledge acquisition (further divided into storage and encoding) and knowledge retrieval. Our curricular design attempted to enhance knowledge acquisition through attention to learner cognitive load (eg, incorporating multiple pauses for discussion), presenting fundamental background concepts, and introducing desirable difficulty by way of a hands-on skills training session. Knowledge acquisition was also promoted through an immediate post-course examination. The curriculum attempted to improve retrieval in the following manners: dispersing content by sending participants pre-course suggested reading material, emphasizing core content and skills in an appropriate context (ie, simulation) and finally through frequent informal testing/quizzing during the course.

The critical importance of the cognitive aspects of modern-day EMR cannot be overemphasized. Advancements in technique have been realized which have led to EMR rivalling even surgery in treatment effectiveness while substantially minimizing risk to patients and cost to healthcare systems.^23^ Meticulous assessment and characterization of polyps is the rate-limiting step guiding the appropriate management of polyps,^24^ including lesion-specific resection strategies.^25,26^ The capacity to determine resectability, estimate the risk of adverse events, and predict the likelihood of lesion recurrence are among the important attributes of a competent EMR practitioner. Although the psychomotor aspect of any “procedural skill” is of obvious importance, possessing a deep understanding of the cognitive knowledge base is a prerequisite for successful LNPCP resection. The complementary nature of knowledge and technical skills in EMR was emphasized throughout the course.

The psychomotor training component of the course was done on a high-fidelity ex-vivo module using a standard endoscope, electrosurgery unit and required disposable devices. Only 3 candidates were engaged in hands-on training at a time, which allowed ample opportunity for discussion and application of the skills taught during the lectures including dynamic injection, snare selection, placement and closure, assessment of the post-EMR defect, clip closure of EMR defects, and snare tip soft coagulation of the margin. We aimed to provide a rich hands-on learning environment that resembled the clinical setting. Narrative data from the qualitative phase of this study suggested this was a desirable aspect of the course. In contrast, learning in larger group settings may be less useful. This barrier is not uncommonly encountered at conferences with live endoscopy training, which may not be optimal to learn a complex skill like piecemeal EMR.

Trainees were excluded from the study. The requisite components of high-quality piecemeal EMR demand higher levels of cognitive processing and skill. Competence in basic colonoscopy and tip control are preconditions for EMR training.^3^ One of the potential contributing factors to the significant improvement in learning observed during the course may have been the limited degree of cognitive fatigue experienced by participants. This was supported by our qualitative data with candidates reporting that the content was appropriately targeted for learners. Trainees mentally occupied by learning other basic skills may face higher levels of cognitive demand, potentially rendering learning ineffective due to increased extraneous processing and limiting germane processing.^20^ Thus, we believe an advanced skill like piecemeal EMR is optimally learned after sufficient competency in basic endoscopy is obtained. General gastroenterology and surgery training programs may not be suitable for learning piecemeal EMR.

Whilst we cannot be certain of the generalizability of our findings, it seems plausible that similarly rigorous short courses in EMR could generate similar results. This is important given the need to upskill large numbers of practicing endoscopists. It is appropriate that endoscopists performing complex resections complete more intensive and lengthy training programs. This format, however, is impractical for most physicians already in independent practice. Moreover, given the continued growth of CRC screening where LNPCPs are being identified more frequently and the rapidly advancing field of endoscopic tissue resection where minimally invasive techniques have become the standard, we need our existing endoscopy workforce to perform much of this work. Highly effective targeted short courses in endoscopic tissue resection may be part of the solution for upskilling endoscopists already in clinical practice.

Although beyond the scope of this study, one might anticipate that widespread participation in focused EMR courses could help address and potentially reverse the concerning trend of increasing surgical resection of non-malignant colorectal polyps.^27^ The reasons for this are unclear, but it is likely that a poor understanding of modern-day endoscopic capabilities and a lack of access to an endoscopist(s) skilled in EMR are contributors. Our results demonstrate that those who completed the EMR course acquired and retained new knowledge and insight on the state-of-the-art management of LNPCPs and became more confident in resecting colorectal polyps in general. Following the course, candidates reported having a greater awareness of the potential of EMR and its limitations. Confidence was a consistent theme in the qualitative data analysis, and this included greater comfort in attempting suitable lesions, along with a better understanding of when to refer to a more experienced endoscopist or surgeon.

Our findings should not imply that participants can become proficient in managing the spectrum of LNPCPs following a course such as ours. Undoubtedly, simply completing an EMR course cannot assure competency. Piecemeal EMR is an advanced technique and outcomes are operator dependent.^28^ Learning a complex skill should be an ongoing process, with frequent and detailed review of skills. It is suggested that learners begin by reviewing the literature and EMR videos. The next step should be to attend a well-designed course for EMR incorporating hands-on training. Finally, one should start to perform the procedure with simpler lesions, ideally under the supervision of a more experienced endoscopist, progressing to more difficult cases. Ongoing feedback and measurement of quality metrics and endpoints are very important to monitor progress.^3^ Currently, there are no firmly established minimum volume targets to achieve competency in EMR, although a recent study that evaluated 6 advanced endoscopy trainees in the United States for EMR competency suggested at least 25 procedures.^29^ It is unclear if fewer cases are acceptable for more experienced endoscopists.

There are several limitations to this study. Candidates who self-identify as women were overrepresented in the study relative to the current demographics of the specialty. The reasons for this are not entirely clear. In addition, participants were self-selecting, which may introduce some bias on their ability to learn a new skill. However, years of experience were balanced. The delayed post-course examination was not invigilated, and therefore we can only presume that the participants followed the given directives (test duration 30 minutes, no access to additional resources). Nevertheless, the imperfect delayed mean test scores suggest that the instructions were adhered to. Although we demonstrated significant knowledge acquisition and retention, the assessment of the psychomotor component was only formative. This form of assessment provides feedback for trainees during training, highlights strengths and weaknesses, and provides points for further improvement, but does not provide an independent, objective measure of psychomotor skills’ achievement. A summative assessment for the psychomotor skills component would require a validated direct observation procedural skills checklist, but such a tool does not currently exist for EMR. Future directives can be aimed at developing a tool to accurately assess a complex skill like EMR, and determine outcomes of critical metrics (eg, complication rate or recurrence rate). Although there are imperfect tools that exist (eg, Direct Observation of Procedural Skills—DOPyS), there is no current evidence that these tools correlate to clinical outcomes.

Another limitation is the qualitative data gathered from only 3 candidates, which is a low number.^16^ However, “code saturation,” which establishes a (heard-it-all) approach, is typically reached with less qualitative data/fewer candidates than “meaning saturation” (understand it all).^16^ Furthermore, it is likely that “code saturation” was reached earlier due to the highly specialized and specific nature of EMR, the homogenous population of candidates (all general gastroenterologists), and iterative sampling. Moreover, the main hypothesis was already established and tested through the quantitative part of the study while the qualitative aspect was explanatory. Hence, concrete code identification and a researcher-driven/deductive approach employing “code saturation” was justified. Recognizing that these findings on the qualitative aspect may not be robust enough to capture all dimensions of a complex procedure like EMR in real-life practice. Lastly, all the participants were volunteer gastroenterologists practicing at tertiary care centers, and thus it is not known if this training activity would have a similar effect on community-based practitioners.

In conclusion, piecemeal EMR is an advanced endoscopic skill of fundamental importance to CRC screening. To derive its full potential and limit unnecessary surgical resection of benign LNPCPs, endoscopists must upskill to become competent. A well-developed curriculum for EMR improves cognitive knowledge and endoscopists report that it improves psychomotor skills and confidence. Further research is needed to validate EMR educational tools and quality metrics for credentialing and assessing competence and outcomes in EMR.

## Supplementary material

Supplementary material is available at *Journal of the Canadian Association of Gastroenterology* online.

gwaf015_Supplementary_Material

gwaf015_Supplementary_Data

## Data Availability

Data are available upon request to the corresponding author.
