# Supplementary material for: Training in endoscopic mucosal resection: effectiveness and clinical utility of a short course for practicing endoscopists
Source: J Can Assoc Gastroenterol. 2025 Jun 21;8(5):184–91. doi: 10.1093/jcag/gwaf015 (PMC12551745; doi:10.1093/jcag/gwaf015)
Supplement: gwaf015_Supplementary_Material [file gwaf015_supplementary_material.docx]

**Supplemental material.1 (Needs assessment)**

Thank you for taking this survey. Generally, EMR as a skill is not systematically learned in general or even most therapeutic Gastroenterology training programs. This survey serves as a needs assessment tool to evaluate what is important and needed by endoscopists in a formal colorectal EMR course.

Please circle a number below based on the importance of each subject.

1. Competence in polyp / lesion evaluation, classification, and assessment of deep invasion risk.

1 ---------> 2 ----------> 3 ----------> 4 ---------> 5 ----------> 6 ----------> 7

Extremely NOT important Extremely important

1. Complex polyp EMR technique (positioning, dynamic injection, snare selection, resection technique, snare tip soft coagulation, and defect evaluation)

1 ---------> 2 ----------> 3 ----------> 4 ---------> 5 ----------> 6 ----------> 7

Extremely NOT important Extremely important

1. Management and Techniques for the “difficult polyp” like ICV and appendiceal orifice lesions

1 ---------> 2 ----------> 3 ----------> 4 ---------> 5 ----------> 6 ----------> 7

Extremely NOT important Extremely important

1. Recognition of post EMR complications and their management (bleeding, perforation, recurrence and residual)

1 ---------> 2 ----------> 3 ----------> 4 ---------> 5 ----------> 6 ----------> 7

Extremely NOT important Extremely important

1. Post EMR surveillance, and recognition and management of recurrence or residual polyp / lesion during surveillance

1 ---------> 2 ----------> 3 ----------> 4 ---------> 5 ----------> 6 ----------> 7

Extremely NOT important Extremely important

1. The ability to train in a safe and controlled environment under supervision, and to perform complex EMR on ex-vivo models.

1 ---------> 2 ----------> 3 ----------> 4 ---------> 5 ----------> 6 ----------> 7

Extremely NOT important Extremely important

1. Recognizing when to biopsy a lesion, or attempt to EMR, or to refer to another endoscopist or a surgeon if the lesion is deemed not endoscopically resectable.

1 ---------> 2 ----------> 3 ----------> 4 ---------> 5 ----------> 6 ----------> 7

Extremely NOT important Extremely important

Thank you,

For any inquiries, please email Ahmed Kayal, MD, ABIM, FRCPC
Email: [ahmed.kayal@ucalgary.ca](mailto:ahmed.kayal@ucalgary.ca)

**Supplemental Material.2 (Model image. Porcine colon model with silicone polyps, Endoscope, and ESU at the ATSSL)**


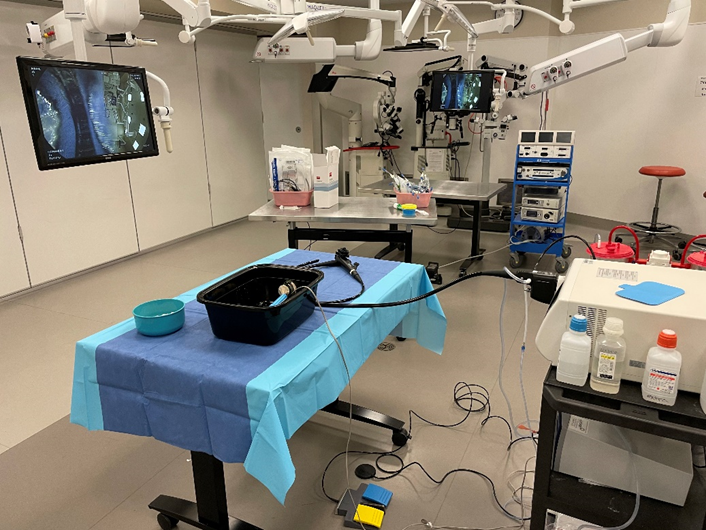


**Supplemental Material.3 (Program objectives and agenda)**

**Objective**

By the end of this course, participants will be able to:

1. Classify the different types of colorectal polyps and recognize their risk of malignancy.
2. Examine the different types of colorectal lesions endoscopically and select the best treatment approach.
3. List the different equipment available to perform an EMR procedure and describe how they are used.
4. Recognize the potential complications of EMR during and after the procedure and explain their management.
5. Demonstrate the basic skill for EMR procedure, which include dynamic injection, resection, defect evaluation, and equipment review.

**Activities**

A few clinical vignettes will be presented and some of the participants will be asked to describe their approach. This is to stimulate discussion between participants and to keep them engaged during the lectures.

**Teaching methods and materials**

Power point presentation and multiple pictures and videos will be used. The videos will show the features of different polyps and resection techniques.

A high Fidelity Ex-vivo colon model with silicon polyps will be used in the simulation lab for hands on training and demonstration of EMR technique.

|  | | The University of Calgary Endoscopic Mucosal Resection Agenda | | | |
| --- | --- | --- | --- | --- | --- |
| Time | Objective | | Slides | Content | **Speaker** |
| 7:30 | - Introduction - Breakfast | | - | - | AK |
| 8:00 | - MCQ testing | |  | - Pre course MCQs |  |
| 8:30 | - Epidemiology of Polyps and Colorectal Cancer | |  | - | AK |
| 9:00 | - Endoscopic Polyp Evaluation | |  | - Kudo pit pattern - Paris classification - Optical enhancements | AK |
| 9:30 | - Risk of submucosal invasion | |  | - Pathology - Location - Polyp features - Role of ESD and surgery | AK  SJH |
| 9:45 | - Activity | | - | - Clinical Vignette | - |
| 10:00 | - Coffee Break | | - | - | - |
| 10:15 | - EMR technique | |  | - Positioning - Dynamic injection and lift - Snare selection and planning of resection - Defect evaluation - Tattoo | AK  SJH |
| 10:45 | - The difficult polyp | |  | - Appendiceal orifice - ICV - Prior resection  - Circumferential resection  - Anorectal junction | AK |
| 11:15 | - Complications and their management of EMR | |  | - Bleeding (intraprocedural and delayed) - Perforation  - Role of prophylactic clipping | AK  SJH |
| 11:45 | - Follow up post EMR | |  | - Recurrence / residual polyp - Post EMR scar evaluation - Surveillance | AK |
| 12:15 | - Activity | |  | - Clinical Vignette |  |
| 12:30 | - MCQ testing | |  | - Post course MCQ |  |
| 13:00 | - Lunch Break | |  |  |  |
| 13:30 | - Hands on training | |  | - Simulation lab | AK  SJH |
| 16:00 | - Activity | |  | - Discussion and feedback |  |
| 16:30 | - Closing remarks | |  |  |  |

**Supplemental Material.4 (Post course survey)**


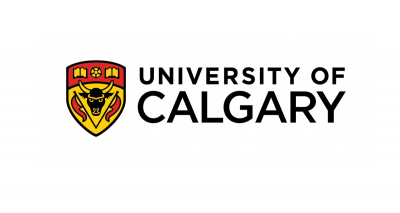


The University of Calgary Endoscopic Mucosal Resection Course
----Post Course Survey----

1. **How cognitively tired were you after the course? (Circle a number below)**

Not tired at all 0 🡪 1 🡪 2 🡪 3 🡪 4 🡪 5 🡪 6 🡪 7 Extremely tired

1. **What is your specialty?**

- Gastroenterology
- Surgery
- Other (please specify)

1. **For how long have you been in practice / performing colonoscopy?**

- Less than 2 years
- 2 to 5 years
- 6 to 10 years
- 11 to 15 years
- More than 15 years

1. **The program met the stated objectives.**

- Strongly Agree
- Agree
- Neutral
- Disagree
- Strongly Disagree

1. **The course was generally useful in terms of knowledge.**

- Strongly Agree
- Agree
- Neutral
- Disagree
- Strongly Disagree

1. **The course was generally useful in terms of technical skills.**

- Strongly Agree
- Agree
- Neutral
- Disagree
- Strongly Disagree

1. **The material and topics were clear and organized.**

- Strongly Agree
- Agree
- Neutral
- Disagree
- Strongly Disagree

1. **I will change / refine my EMR technique after this course.**

- Strongly Agree
- Agree
- Neutral
- Disagree
- Strongly Disagree

1. **The EMR model was very similar to a real polyp.**

- Strongly Agree
- Agree
- Neutral
- Disagree
- Strongly Disagree

1. **In terms of course delivery, I prefer the whole material in 1 day as it was delivered and not over 2 or 3 days.**

- Strongly Agree
- Agree
- Neutral
- Disagree
- Strongly Disagree

1. **I will be participating in future University of Calgary EMR courses for updates on EMR.**

- Very likely
- Likely
- Neutral
- Unlikely
- Very unlikely

1. **How often do you perform colorectal EMR of lesions larger than 2cm in your practice?**

- I do not perform complex EMR
- 1 to 2 per month
- 3 to 5 per month
- 6 to 10 per month
- More than 10 per month

1. **What are the 3 things you would change or add in future courses?**


1. **What are the 3 things you liked most about this course?**


End of Survey

**Supplemental Material.5 (Reflexivity statements)**

- **KM**

KM is a Nephrologist and an International Medical Graduate who completed postgraduate specialty training in the United Kingdom. Following completion of post-graduate medical training, he completed a PhD in educational psychology. He is now a Professor in the Department of Medicine and his teaching and research activities are largely in undergraduate medical education, although he previously served and the Program Director for Nephrology Residency Training Program at the Cumming School of Medicine and is currently the Academic Advisor in this program. His clinical focus is as a nephrologist involved in the care of kidney transplant patients, and his education focus is in the areas of assessment, remediation, cognition, and curriculum design. He is involved in both quantitative and qualitative research in medical education.

- **AK**

AK is a Gastroenterologist with subspecialty training in advanced therapeutic endoscopy. He completed Internal medicine and Gastroenterology training at the University of British Columbia in Vancouver, BC. He then completed subspecialty training at the University of Calgary and practicing as a Gastroenterologist. AK also completed a Master’s degree in Health Science – Medical education (Msc) from the Department of Community Health Sciences, University of Calgary. His clinical focus as a gastroenterologist involves pancreatico-biliary endoscopy, endoscopic ultrasound, and colo-rectal endoscopic mucosal resection. His education focus are in areas of endoscopy training, residents’ education, educational psychology, and curriculum design. He is currently involved in clinical research in the Division of Gastroenterology and Hepatology, University of Calgary.

SUPPLEMENTAL MATERIAL 6 (Blue print and MPL scores)

| **Content area** | **Specific topics** | **Weight (%/#)** | **Task evaluated (#)** | |
| --- | --- | --- | --- | --- |
|  |  |  | Recognition or Recall | Application or Critical thinking |
| Evaluation | - SMI risk assessment - Lesion description - Alternative approaches | 25% / 6 | 3 | 3 |
| EMR procedure | - Position optimization - Dynamic injection and lift - En-bloc Vs piecemeal resection - STSC and CAST - Defect evaluation - Equipment | 30% / 8 | 3 | 5 |
| Complications | - Bleeding (IPB 7 CSPEB) - Risk assessment of complications - Perforation - Management of complications - Delayed complications | 30% / 8 | 4 | 4 |
| Follow up | - RRA evaluation and management - Scar assessment - Surveillance and further management | 15% / 4 | 2 | 2 |

**MPL for all items based on the modified Nedelsky’s method**

| MCQ I | Elimination probability of items by minimally competent candidates | Minimum performance level (MPL) = |
| --- | --- | --- |
| 1 | AK: A= 90% B= 60% D= 60% SC: A= 100% B= 50% D= 50% | AK: 0.53 SC: 0.50 |
| 2 | AK: B= 60% C= 75% D= 90% SC: B= 50% C= 80% D= 100% | AK: 0.57 SC: 0.59 |
| 3 | AK: A= 70% B= 50% C=90% SC: A= 80% B= 50% C=80% | AK: 0.53 SC: 0.53 |
| 4 | AK: A= 25% C= 25% D= 50% SC: A= 40% C= 80% D= 50% | AK: 0.33 SC: 0.43 |
| 5 | AK: A= 80% C= 70% D= 40% SC: A= 80% C= 50% D= 30% | AK: 0.48 SC: 0.42 |
| 6 | AK: A= 75% B= 75% C= 75% SC: A= 50% B= 50% C= 50% | AK: 0.57 SC: 0.40 |
| 7 | AK: A= 80% B= 70% C= 60% SC: A= 80% B= 80% C= 80% | AK: 0.53 SC: 0.63 |
| 8 | AK: A= 90% B= 50% D= 70% SC: A= 100% B= 80% D= 50% | AK: 0.53 SC: 0.59 |
| 9 | AK: B= 40% C= 30% D= 50% SC: B= 35% C= 35% D= 35% | AK: 0.36 SC: 0.34 |
| 10 | AK: A= 70% C= 40% D= 40% SC: A= 60% C= 50% D= 30% | AK: 0.40 SC: 0.42 |
| 11 | AK: A= 80% B= 70% C= 50% SC: A= 80% B= 80% C= 80% | AK: 0.50 SC: 0.63 |
| 12 | AK: A= 40% B= 50% C= 70% SC: A= 70% B= 40% C= 40% | AK: 0.42 SC: 0.40 |
| 13 | AK: A= 50% B= 30% C= 20% SC: A= 70% B= 50% C= 40% | AK: 0.33 SC: 0.42 |
| 14 | AK: B= 30% C= 60% D= 60% SC: B= 30% C= 70% D= 70% | AK: 0.40 SC: 0.43 |
| 15 | AK: A= 20% C= 10% D= 30% SC: A= 30% C= 50% D= 30% | AK: 0.29 SC: 0.34 |
| 16 | AK: B= 80% C= 50% D= 40% SC: B= 80% C= 60% D= 60% | AK: 0.44 SC: 0.50 |
| 17 | AK: B= 30% C= 40% D= 60% SC: B= 30% C= 50% D= 80% | AK: 0.37 SC: 0.42 |
| 18 | AK: A= 50% B= 50% C= 50% SC: A= 80% B= 70% C= 50% | AK: 0.40 SC: 0.50 |
| 19 | AK: A= 100% C= 80% D= 80% SC: A= 90% C= 50% D= 80% | AK: 0.71 SC: 0.56 |
| 20 | AK: A= 40% B= 10% D= 10% SC: A= 80% B= 50% D= 30% | AK: 0.29 SC: 0.42 |
| 21 | AK: A= 40% B= 50% D= 60% SC: A= 30% B= 30% D= 30% | AK: 0.40 SC: 0.32 |
| 22 | AK: A= 50% C= 70% D= 30% SC: A= 50% C= 50% D= 50% | AK: 0.40 SC: 0.40 |
| 23 | AK: A= 30% B= 30% D= 20% SC: A= 30% B= 30% D= 30% | AK: 0.31 SC: 0.32 |
| 24 | AK: A= 20% C= 20% D= 40% SC: A= 30% C= 30% D= 30% | AK: 0.31 SC: 0.32 |
| 25 | AK: B= 50% C= 50% D= 50% SC: B= 30% C= 30% D= 60% | AK: 0.40 SC: 0.36 |
| 26 | AK: A= 20% C= 40% D= 80% SC: A= 30% C= 30% D= 50% | AK: 0.39 SC: 0.34 |
| TOTAL MPL= | | AK: 11.19 SC: 11.48 |
| MCQ II | Elimination probability of items by minimally competent candidates | Minimum performance level (MPL) = |
| 1 | AK: B= 50% C= 60% D= 90% SC: B= 50% C= 80% D= 100% | AK: 0.50 SC: 0.59 |
| 2 | AK: A= 50% B= 80% C= 80% SC: A= 50% B= 50% C= 80% | AK: 0.53 SC: 0.45 |
| 3 | AK: A= 80% C= 50% D= 80% SC: A= 80% C= 50% D= 80% | AK: 0.53 SC: 0.53 |
| 4 | AK: A= 30% C= 50% D= 40% SC: A= 40% C= 50% D= 80% | AK: 0.36 SC: 0.43 |
| 5 | AK: A=20% C= 40% D= 80% SC: A= 40% C= 40% D= 60% | AK: 0.39 SC: 0.38 |
| 6 | AK: A= 50% B= 50% D= 20% SC: A= 80% B= 100% D= 50% | AK: 0.36 SC:0.58 |
| 7 | AK: B= 90% C= 90% D= 90% SC: A= 40% C= 40% D= 60% | AK: 0.77 SC: 0.38 |
| 8 | AK: A= 100% B= 50% D= 70% SC: A= 60% B= 40% D= 40% | AK: 0.56 SC: 0.38 |
| 9 | AK: B= 40% C= 30% D= 50% SC: A= 50% C= 50% D= 50% | AK: 0.36 SC: 0.40 |
| 10 | AK: A= 100% B= 80% C= 50% SC: A= 80% B= 80% C= 50% | AK: 0.59 SC: 0.53 |
| 11 | AK: B= 70% C= 50% D= 80% SC: B= 80% C= 80% D= 60% | AK: 0.50 SC: 0.56 |
| 12 | AK: A= 80% B= 30% D= 80% SC: A= 50% B= 80% D= 60% | AK: 0.47 SC: 0.48 |
| 13 | AK: A= 60% B= 30% C= 10% SC: A= 70% B= 50% C= 40% | AK: 0.33 SC: 0.42 |
| 14 | AK: A= 70% C= 60% D= 80% SC: A= 80% C= 80% D= 80% | AK: 0.52 SC: 0.63 |
| 15 | AK: B= 80% C= 50% D= 40% SC: B= 60% C= 60% D= 60% | AK: 0.44 SC: 0.45 |
| 16 | AK: A= 20% C= 10% D= 30% SC: A= 30% C= 50% D= 30% | AK: 0.29 SC: 0.34 |
| 17 | AK: B= 80% C= 50% D= 40% SC: B= 80% C= 60% D= 60% | AK: 0.43 SC: 0.50 |
| 18 | AK: A= 50% B= 50% D= 50% SC: A= 80% B= 40% D= 40% | AK: 0.40 SC:0.42 |
| 19 | AK: B= 80% C= 100% D= 80% SC: B= 50% C= 50% D= 90% | AK: 0.71 SC: 0.48 |
| 20 | AK: B= 40% C= 10% D= 10% SC: B= 30% C= 50% D= 80% | AK: 0.29 SC: 0.42 |
| 21 | AK: A= 50% B= 50% C= 50% SC: A= 50% B= 50% C= 30% | AK: 0.40 SC: 0.37 |
| 22 | AK: A= 80% C= 50% D= 50% SC: A= 30% C= 70% D= 50% | AK: 0.46 SC: 0.40 |
| 23 | AK: A= 20% B= 30% D= 30% SC: A= 40% B= 60% D= 30% | AK: 0.31 SC: 0.37 |
| 24 | AK: A= 20% B= 40% C= 20% SC: A= 50% B= 50% C= 100% | AK: 0.31 SC: 0.50 |
| 25 | AK: A= 50% C= 50% D=50% SC: A= 30% C= 30% D= 40% | AK: 0.40 SC: 0.33 |
| 26 | AK: A= 40% B= 40% D= 70% SC: A= 50% B= 60% D= 60% | AK: 0.40 SC: 0.43 |
| Total MPL= | | AK: 11.61 SC: 11.75 |

Pre- and delayed MCQs

1. Non-granular laterally spreading lesions have an overall risk of submucosal invasive cancer of
   A- <2%
   B- 5-9%
   C- 10-15%
   D- 16-20%
2. According to a recent randomized controlled trial, using a colloid solution for EMR was found to be
   A- Superior to normal saline requiring significantly less resections, less injections, and overall less EMR time
   B- Superior to normal saline requiring less injections but similar number of resections and overall EMR time.
   C- No difference in number of injections or resections, and it is generally an endoscopist preference
   D- Inferior to normal saline and requires a greater number of injections and resections and overall more EMR time.
3. Based on the picture shown, how would you describe this lesion?
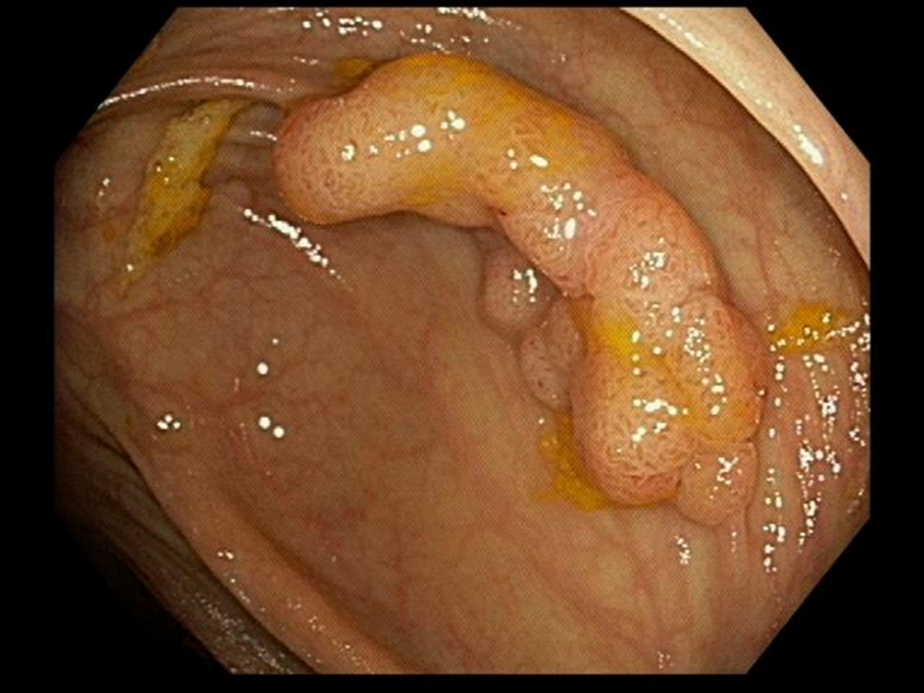

   A- Granular LSL, Paris 0-IIa + Is Kudo IV
   B- Non granular LSL, Paris 0-IIa Kudo II
   C- Granular LSL, Paris 0-IIb Kudo IV
   D- Non granular LSL, Paris 0-IIa + c, Kudo V
4. Based on the picture shown of a RECTAL polyp, what is the recommended next step in management?

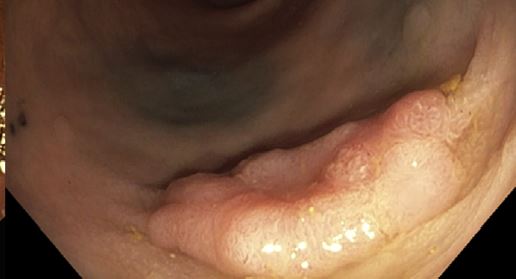
 Klein, A. Gastroint Endoscopy (2015)
   A- Refer the patient for EMR
   B- Refer the patient for ESD or TAMIS
   C- Refer the patient for surgery + creation of a stoma
   D- Obtain biopsies first then decide next step of management.
5. You are asked to assess a patient for consideration of EMR. A 68-year-old man with a history of coronary artery disease, chronic kidney disease, and Diabetes Miletus. A 3cm granular LSL was found in the peri appendiceal orifice area. It occupies around 40% of the circumference of the orifice. It a Paris 0-IIa + Is and Kudo III. The proximal edge is difficult to see inside the orifice, but the distal edge is clearly visible in the cecum.
   Based on this information, what would make the EMR of this lesion less likely to be successful?
   A- Comorbidities of the patient
   B- The proximal edge being inside the orifice
   C- The Paris classification and the Kudo pit pattern
   D- The involvement of 40% of the orifice.
6. Which of the following sites increases the risk of deep submucosal invasion of LSL?
   A- Cecum
   B- Ascending colon
   C- Transverse colon
   D- Rectum.
7. To decrease the risk of recurrence after piece meal endoscopic mucosal resection, which of the following is the recommended technique?

A- Widefield resection of normal mucosa

B- Non-contact thermal treatment of small left-over islands and defect edges (like Argon Plasma Coagulation)

C- Use of cautery with snare resections

D- Snare tip soft coagulation of the defect edges.

1. What is the general size limit of colonic polyps for an en-bloc resection to decrease risk of complications?
   A- 10-14mm
   B- 14-19mm
   C- 20-24mm
   D- 25-30mm
2. The use of thin wired (0.3mm monofilament) snares can be helpful in
   A- Poorly lifting lesions
   B- Polyps in the cecum
   C- Patients with an increased risk of bleeding
   D- Large sessile polyps
3. A patient was referred to you for a “difficult EMR procedure”. It was attempted at a peripheral hospital, but the lesion could not be resected completely due to its difficult location at the hepatic flexure. The lesion is granular, Paris 0-IIa and was around 3.5cm in size. You were asked to assess and consider completing the EMR.
   Due to scarring, the lesion poorly lifts with injection and it is difficult to catch the tethered fibrotic area with a snare. What is your next step for EMR?
   A- Repeat the procedure 4 weeks later and attempt EMR then.
   B- Use cold avulsion with biopsy forceps and snare tip soft coagulation technique (CAST)
   C- Refer for ESD
   D- Use of hot avulsion technique to apply some cautery effect with resection of fibrotic areas, which decreases risk of bleeding and residual adenoma.
4. Which of the following is known to be associated with an increased risk of residual or recurrent adenoma post EMR?
   A- Lesions larger than 10mm in size
   B- The presence of vessels in the EMR defect
   C- Intraprocedural perforation
   D- Lesions with high grade dysplasia
5. A large polyp was resected with EMR in the sigmoid colon. No intraprocedural or post resection bleeding is noted. However, there were large 3-4mm visible vessels in the defect. What is the next step?
   A- Ablate the vessels with snare tip soft coagulation.
   B- Clip the vessels.
   C- Close the defect with clips.
   D- No intervention.
6. A patient is having a repeat colonoscopy for post EMR site check at 6 months (SC1). The lesion was a 50mm LSL in the ascending colon. It was a granular, Paris 0-IIa with Kudo IV. The resection was complicated by intraprocedural bleeding that was treated with snare tip soft coagulation. Final pathology showed a tubular adenoma with no high-grade dysplasia.
   Based on the Sydney EMR recurrence tool (SERT) score, this lesion is considered;
   A- Low risk of recurrence
   B- moderate risk of recurrence
   C- High risk of recurrence
   D- Cannot estimate risk of recurrence
7. Having a strategy before commencing EMR of a large polyp in the colon is important. A colleague in the next endoscopy room asked you to “quickly peek” at a polyp before ongoing resection and asked you for advice. You see the lesion is positioned at 6 O’clock, there was a pool of fluid obscuring part of the polyp. You see there was a part resected from the proximal end of the polyp, and another part at the distal end behind a fold that was also resected. The 2 resections seem far apart with most of the polyp present in between.
   What would you suggest for planning to optimize EMR?
   A- Reposition the patient to shift the pool of fluid away from the lesion, and start working at the distal end behind the fold given that this is the “least accessible area”
   B- Reposition the patient to shift the pool of fluid away from the lesion, and start working at the proximal end given that this is the “most accessible area”
   C- Dynamic injection should be performed with lifting of the polyp away from the scope but towards the lumen.
   D- Dynamic injection should be performed with lifting the polyp proximally and away from the center of the lumen but towards the scope.
8. A patient known to you presents to the ED with abdominal pain. An EMR of a large polyp in the transverse colon was performed 2 days prior. It was a 35mm non-granular LSL. Paris 0-IIa +Is with Kudo IVL. Some intraprocedural bleeding was encountered and was managed effectively with STSC (snare tip soft coag) with good hemostasis. Limited dye injection was used due to shortage, but the post EMR defect appeared intact (thought to be Sydney type 1).
   The patient is slightly tachycardic at 114bpm. Other vitals are normal. Abdominal exam shows a diffusely tender abdomen and the patient is in moderate – severe discomfort. An abdominal X-ray was arranged. There is evidence of free air and the patient was admitted for further management.
   Which of the above mentioned features is associated with an increased risk of perforation post EMR?
   A- The size of the lesion
   B- The location of the lesion
   C- The intraprocedural bleeding encountered.
   D- The use of limited dye during lifting of the lesion
9. Which of the following is associated with a risk of clinically significant post EMR bleeding?
   A- intraprocedural bleeding during EMR
   B- Use of electrosurgical current controlled by a micro-processor unit.
   C- Distal location of the lesion in the colon
   D- LSL / flat Paris morphology (0-IIa or 0-IIb)
10. You are performing a complex EMR of a large polyp in the Transverse colon. The lesion is a 40mm LSL. It is granular with a Paris 0-IIa morphology. You notice that despite good lifting of the lesion with dynamic injection, the snare with cautery resection needed around 5 to 7 seconds to cut through a large piece of the polyp. You use the catheter with a retracted needle and inject dye in the defect. A defect area stains blue but there is a round area of white tissue that does not absorb the dye.
    What is the next best step in management?


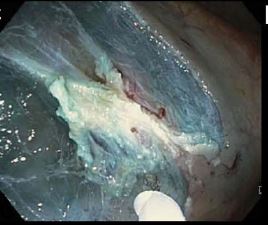

A- Clip closure for the white area (does not stain blue) and finish the EMR procedure, and monitor the patient afterwards.
B- Abort the procedure and obtain an urgent surgical consultation to plan surgery.
C- Immediate closure of the defect with clips, and admit the patient for monitoring.
D- Continue the EMR procedure and no need for other intervention.

1. Based on the picture shown. What is the post EMR Sydney classification of deep mural injury?

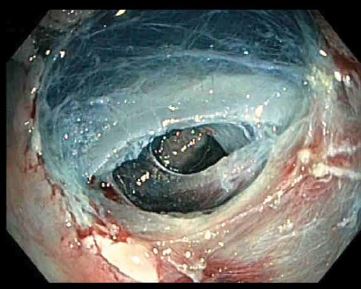

   A- Type I
   B- Type II
   C- Type III
   D- Type IV
2. An 87-year-old man presented to the ED 9 days post EMR of a large polyp in the cecum with multiple episodes of bright red blood per rectum. The lesion was 45mm in size and was a Paris 0-IIa and granular. The lesion was lifted using a mixture of a colloid solution, dye, and epinephrine (1: 100,000). No endoscopic closure was performed, and the defect showed no evidence of deep mural injury. The patient is slightly tachycardic and feels light-headed. Other vitals are all normal and his mentation is normal. Hemoglobin at presentation is at 105 g/L compared to 135 prior to the procedure. His Past medical history is significant for Coronary artery disease, hypertension, and a history of prostate cancer that was treated 12 years ago. His medications include Aspirin, Amlodipine, Ramipril, and Atorvastatin. The patient was managed in ED. A colonoscopy shows the large EMR defect with a visible vessel noted.
   what is your next step in management?
   A- No intervention giving that there is no active bleeding.
   B- Dual therapy with epi injection and placement of an Endoclip around the vessel.
   C- Monotherapy with placement of an Endoclip or BICAP therapy.
   D- Endoscopic treatment should be avoided giving the recent EMR and the risk of perforation associated. Referral to interventional radiology for selective embolization should be done.
3. For the above patient, the clinically significant post EMR bleeding (CSPEB) risk based on the Australian colonic EMR study (ACE) is ;
   A- Low risk
   B- Intermediate risk
   C- High risk
   D- Cannot determine the risk
4. What is the reason that prophylactic clipping for ALL EMR defects to decrease the risk of post procedure bleeding is not generally adopted?
   A- Recent evidence showed mixed results, and it does not seem to be cost effective
   B- Recent evidence showed that this strategy may be associated with an increased risk of immediate complications.
   C- Recent evidence showed that this strategy increased procedure time significantly with no significant decrease in delayed bleeding risk.
   D- Recent evidence showed that this strategy increased procedure time, but decreased bleeding risk significantly and should be considered for all post EMR defects.
5. EMR of a complete circumferential laterally spreading lesion of the colon is associated with
   A- Significant fibrosis and difficult to lift
   B- Clinically significant stricturing, and should be treated with endoscopic dilation
   C- High risk of residual / recurrent adenoma (RRA) at first, second, and third surveillance colonoscopy despite treatment of RRA.
   D- High risk of immediate complications post EMR.

1. Endoscopic examination of a post EMR scar is a critical follow up component to recognize residual / recurrent adenoma. Studies have shown that NBI examination is superior than HD-WL examination in which of the following situations?
   A- Large post EMR scars of > 30mm
   B- If the specimen resected showed HGD or invasive cancer
   C- Flat lesions where a transition of non-neoplastic kudo pit pattern to neoplastic pattern is noted.
   D- Elevated small scars / polyps to differentiate clip artifacts from true residual or recurrent adenoma.
2. The first surveillance colonoscopy (SC1) is recommended at 4-6 months post EMR. In which of the following scenarios the risk of RRA is the least?
   A- 50mm granular lesion. No intraprocedural bleeding during EMR, Sydney type 1 defect post EMR defect, and histopathology showing tubular adenoma with no HGD.
   B- 30mm non-granular polyp. No intraprocedural bleeding during EMR, Sydney type 1 post EMR defect, and histopathology showing tubular adenoma with no HGD.
   C- 25mm granular polyp. Mild intraprocedural bleeding during EMR treated successfully and complete resection achieved. Sydney type 0 post EMR defect, and histopathology showing tubular adenoma with no HGD.
   D- 25mm granular polyp. No intraprocedural bleeding encountered. Sydney type II post EMR defect, and histopathology showing tubular adenoma with focal HGD with negative margins.
3. A post EMR scar is being evaluated. Non-targeted large area biopsies to assess for adenomatous lesions in the scar is associated with
   A- Increased risk of false negative
   B- Increased risk of false positive
   C- Increased risk of bleeding
   D- Increased risk of fibrosis and scar artifact
4. Residual / recurrent adenoma detected during surveillance colonoscopy post EMR should be treated with
   A- Cold avulsion + snare tip coagulation (CAST) followed by snare resection if not able to.
   B- Snare resection followed by CAST if not able to
   C- CAST followed by thermal ablation if not able to
   D- Snare resection followed by thermal ablation if not able to

Post Test MCQs

1. Granular laterally spreading lesions in the ascending colon have an overall submucosal invasive cancer risk of (Paris 0-IIa)
   A- 2-3%
   B- 4-5%
   C- 6-10%
   D- >10%
2. The use of dye (methylene blue or indigo carmine) while performing EMR is suggested because:
   A- It provides extra protection and decreases risk of submucosal injury during EMR.
   B- Dynamic injection and lifting tend to be easier and lasts longer before the submucosal lift dissipates.
   C- Decreases risk of intra-procedural bleeding
   D- Delineates the margins of the polyp and it makes it easier to recognize the muscularis propria post resection.
3. Based on the picture shown, and in addition to size and location, how would you describe this lesion?


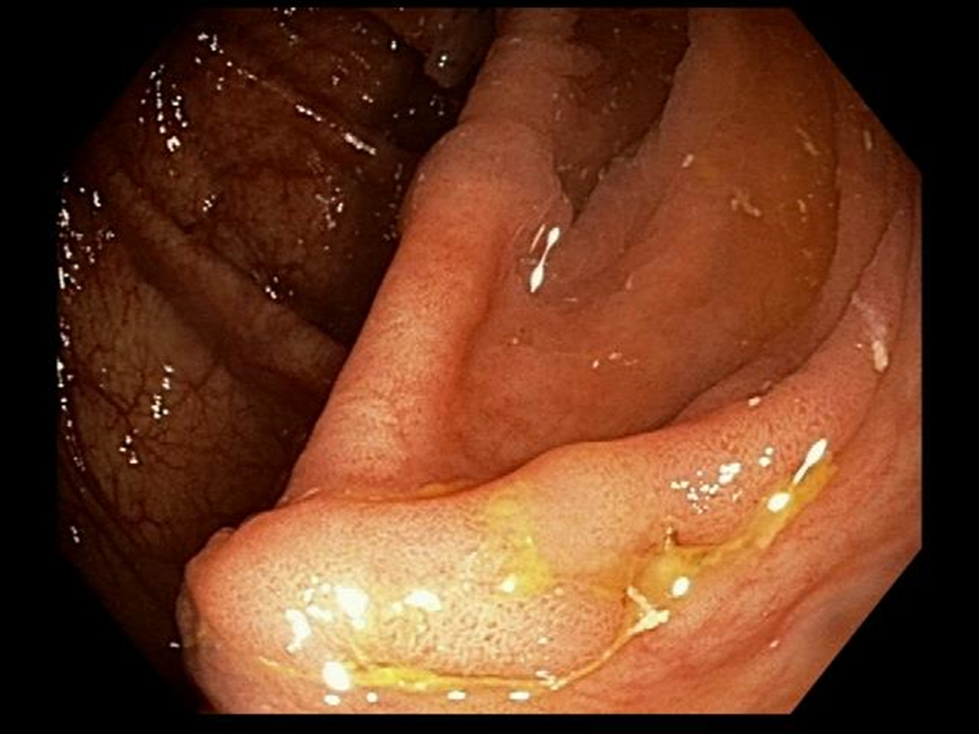

A- Granular LSL, Paris 0-IIa + Is Kudo IV
B- Non granular LSL, Paris 0-IIa Kudo II
C- Granular LSL, Paris 0-IIb Kudo III
D- Non granular LSL, Paris 0-IIa + c, Kudo V

1. What is the best management for a polyp that is involving 75% of the appendiceal orifice? The polyp is described as granular 3cm Paris 0-IIa with Kudo III pit pattern
   A- Refer for EMR
   B- Refer for cecectomy.
   C- Refer for ESD
   D- Perform vigorous biopsies to exclude cancer
2. You were asked to assess a patient for a second opinion. A healthy 53-year-old man was found to have a laterally spreading, non-granular lesion involving the ICV. The polyp seems to extend into the TI. Which of the following is not helpful in evaluating this lesion?
   A- Use of an Endocuff
   B- Use of a distal attachment cap
   C- Use of optical enhancement to differentiate the polyp pits from ileal villi.
   D- Use of a slim colonoscope
3. Which of the following lesions is associated with the most increased risk of deep submucosal invasion?
   A- Paris 0-IIa
   B- Paris 0-IIc
   C- Paris 0-IIa + Is
   D- Paris 0-IIb + Is
4. Regarding snare tip soft coagulation (STSC) of the defect edges post EMR, which of the following must be met to apply this technique?
   A- All visible polyp must be resected before STSC
   B- Polyp islands in the defect can be treated with STSC, but polyp in the edges must be resected.
   C- Polyp on the edges of the defect can be treated by STSC, but polyp islands must be resected.
   D- Small lesions post EMR, less then 4mm, can be treated by STSC regardless.
5. Attempted en-block resection of lesions greater than 30mm is strongly associated with
   A- Recurrence
   B- Visible and raised scar at site of resection.
   C- Deep submucosal / muscle injury
   D- Delayed bleeding.
6. The use of thin wired (0.3mm monofilament) snares can be helpful in
   A- Lesions at the Appendiceal orifice.
   B- Polyps in the cecum
   C- Patients with an increased risk of bleeding
   D- Large sessile polyps
7. A patient was referred to you for a “difficult EMR procedure”. It was attempted at a peripheral hospital, but the resection was not complete due to its difficult location. Biopsies were taken prior to this attempt as well (path: sessile serrated adenoma). The lesion was marked with tattoo and sent to you for further evaluation. You note the lesion in the hepatic flexure and the tattoo is involving the base of the lesion. You attempt to lift the lesion but fail due to fibrosis and tethering. Which of the following options is associated with “poor lifting sign”
   A- Histopathology and location.
   B- Tattoo injection technique, and histopathology of the lesion
   C- Previous EMR attempt, previous biopsies, and location of the lesion
   D- Tattoo injection technique, Previous EMR attempt and biopsies
8. Which of the following is known to be associated with an increased risk of residual or recurrent adenoma post EMR?
   A- Lesions larger than 40mm in size
   B- The presence of vessels in the EMR defect
   C- Intraprocedural perforation
   D- Sessile serrated and traditional serrated adenoma.
9. You experience a significant intraprocedural bleeding event during EMR. You inject some epi around the area to slow down the bleeding. You follow with soft tip coagulation, but the bleeding is persistent and not controlled. The EMR procedure is not complete and residual polyp remains. What is your next best step in management?
   A- Attempt to switch to forced coagulation at the electro cautery unit.
   B- Attempt to clip the bleeding site, and attempt to complete EMR
   C- Attempt to use a coagulation grasper to cauterize the vessel, and attempt to complete the EMR
   D- Apply hemo spray and complete the resection after 2 weeks.
10. A patient is having a repeat colonoscopy for post EMR site check at 6 months (SR1). The lesion was a 50mm LSL in the ascending colon. It was a granular, Paris 0-IIa with Kudo IV. The resection was complicated by intraprocedural bleeding that was treated with snare tip soft coagulation. Final pathology showed a tubular adenoma with positive high-grade dysplasia.
    what are the components mentioned that would increase risk of recurrence based on the SERT score?
    A- Location in the ascending colon, high grade dysplasia, Paris classification
    B- Location in the ascending colon, intraprocedural bleeding, high grade dysplasia
    C- size of the lesion, Paris classification, high grade dysplasia
    D- High grade dysplasia, intraprocedural bleeding, size of the lesion

1. To optimize lifting of lesions. A good dynamic injection technique should be used. Which of the following make dynamic injection less effective?
   A- Aim to elevate the lesion towards the scope and into the lumen.
   B- Use of 22g needle or larger size for optimal injection.
   C- Mix the solution with 1:100,000 epi to keep a clean and dry EMR field during the procedure.
   D- Use of a colloid solution rather than saline (crystalloid)
2. Which of the following is associated with a risk of clinically significant post EMR bleeding?
   A- High grade dysplasia on histopathology
   B- Use of electrosurgical current controlled by a micro-processor unit.
   C- Distal location of the lesion in the colon
   D- LSL / flat Paris morphology (0-IIa or 0-IIb)
3. You were called about a patient who presented to ED with abdominal pain. Your colleague performed an EMR procedure few days ago. The lesion resected was a 30mm Paris 0-Iia + Is in the Sigmoid colon. It was resected piecemeal successfully, and the defect thought to be DMI type II. There was a shortage of Methylene blue, so no extra dye was used to interrogate all of the defect. Some intraprocedural bleeding was treated successfully with STSC. The lesion was a tubulovillous adenoma with high grade dysplasia on histopathology. The patient presents with abdominal pain and an AXR shows mild but positive free air under the diaphragm. The patient was admitted for further evaluation.
   Which of the following features is associated with an increased risk of perforation post EMR?
   A- The size of the lesion
   B- The presence of high grade dysplasia on histopathology
   C- The intraprocedural bleeding encountered.
   D- The use of limited dye during lifting of the lesion
4. EMR of large polyps at which site of the colon is associated with an increased risk of post EMR deep submucosal injury / perforation?
   A- Transverse colon
   B- Cecum
   C- Ascending colon
   D- Sigmoid colon
5. What is the post EMR Sydney classification of deep mural injury of the classic “target sign”?
   A- Type I
   B- Type II
   C- Type III
   D- Type IV

1. A 67-year-old man presented to the ED 2 days post EMR of a large polyp in the Descending colon. He has had several episodes of bright red blood per rectum. The lesion was 45mm in size and was a Paris 0-IIa and granular. The lesion was lifted using a mixture of a colloid solution and dye. No Epinephrine was used in the injectate. No endoscopic closure was performed, and the defect showed no evidence of deep mural injury. The patient is slightly tachycardic but otherwise well. Other vitals are all normal and his mentation is normal. Hemoglobin at presentation is at 127 g/L compared to 135 prior to the procedure. His Past medical history is significant for hypertension and osteoarthritis. His medications include Amlodipine, Ramipril, and PRN Advil. The patient was managed in ED. A colonoscopy showed the large EMR defect with an adherent clot, and only a hemosiderin spot was noted after irrigation of the clot.
   what is your next step in management?
   A- No intervention giving that there is no active bleeding and no high risk feature noted.
   B- Dual therapy with epi injection and placement of an Endoclip around the hemosiderin spot.
   C- Monotherapy with placement of an Endoclip or BICAP therapy.
   D- Endoscopic treatment should be avoided giving the recent EMR and the risk of perforation associated. Referral to interventional radiology for selective embolization should be done.
2. For the above patient, the clinically significant post EMR bleeding (CSPEB) risk score based on the Australian colonic EMR study (ACE) is
   A- 2 points
   B- 3 points
   C- 5 points
   D- 6 points
3. In which of the following situations clip closure of post EMR defects can be considered to decrease risk of delayed bleeding?
   A- partial closure of distal defects.
   B- Complete closure of distal defects
   C- Partial closure of proximal defects
   D- Complete closure of proximal defects
4. A patient was referred to you for evaluation of a difficult EMR procedure. You find a large LSL involving the ICV. You examine the lesion closely and you find both lips of the valve are involved. It was a non-granular, Paris 0-IIa +Is lesion and around 40-50mm in size and occupies half the circumference of the cecum.
   Based on the above, which feature is known to be associated with an increased risk of EMR failure and RRA?
   A- The location of the polyp in the cecum.
   B- The involvement of both lips of the ICV
   C- The non-granular morphology
   D- The size of the polyp
5. You are evaluating a patient who had an EMR of a large polyp 6 months ago. The lesion was a 40mm granular polyp in the ascending colon. The defect was closed with clips post resection. You are evaluating the scar and notice few nodular elevations of the mucosa along the scar. What is the best next step of management?
   A- These are likely clip artifacts and they need no further treatment.
   B- Obtain random biopsies of the whole scar and schedule another colonoscopy for resection if histopathology shows adenomatous tissue.
   C- Apply NBI if available and evaluate for the presence of neoplastic pit pattern to obtain targeted biopsies.
   D- Resect all nodular elevations seen on scar evaluation.
6. You completed an EMR procedure few days ago and got the histopathology result of tubular adenoma with no HGD. The lesion was a 30mm granular LSL in the ascending colon. No intraprocedural bleeding was encountered and the post EMR defect shows a type I DMI. When is the best time to book the patient for their first scar assessment post EMR (SC1)?
   A- 6 months post procedure.
   B- 12 months post procedure
   C- 18 months post procedure
   D- 3 months post procedure
7. A polyp was resected with EMR 6 months ago. The standard of care is to biopsy the scar / elevated nodular mucosa to rule out recurrence. However, scrupulous evaluation and the use of NBI is suggested to
   A- Avoid biopsy if possible and make an optical diagnosis
   B- Decrease false negative rate with targeted biopsies
   C- Decrease false positive rate with targeted biopsies
   D- Increase true positive rates with targeted biopsies
8. Residual / recurrent adenoma detected during surveillance colonoscopy post EMR should be treated with snare resection as first line. However, this may be challenging because
   A- There is an increased risk of bleeding with snare resection of RAA
   B- There is an increased risk of perforation with snare resection of RAA
   C- The fibrosis and tethering of the submucosa secondary to previously treated lesions make ”snare capture” difficult.
   D- The fibrosis and tethering of the submucosa secondary to previously treated lesions make other utilities like CAST more effective than snare resection, even if good capture with the snare is achieved.

MCQs Key Answers

| Pre-test key answers | Post-test key answers |
| --- | --- |
| 1. C  2. A  3. A  4. B  5. B  6. D  7. D  8. B  9. A  10. B  11. D  12. D  13. C  14. A  15. B  16. A  17. A  18. D  19. B  20. C  21. A  22. B  23. C  24. B  25. A  26. B | 1. A  2. D  3. B  4. B  5. B  6. B  7. A  8. C  9. A  10. D  11. A  12. C  13. D  14. B  15. A  16. B  17. A  18. C  19. A  20. A  21. B  22. B  23. C  24. A  25. B  26. C |

References for answers

• Klein, A., & Bourke, M. J. (2017). How to perform high-quality endoscopic mucosal resection during colonoscopy. Gastroenterology, 152(3), 466-471.

• Burgess, N. G., Hourigan, L. F., Brown, G. J., Singh, R., Raftopoulos, S. C., Moss, A., ... & Bourke, M. J. (2014). Tu1481 gross morphology and lesion location stratify the risk of invasive disease in advanced mucosal neoplasia of the colon: results from a large multicenter cohort. Gastrointestinal Endoscopy, 79(5), AB556.

• Bourke, M. J., & Heitman, S. J. (2019). Endoscopic Mucosal Resection and Endoscopic Submucosal Dissection Are Complementary in the Treatment of Colorectal Neoplasia. Clinical Gastroenterology and Hepatology, 17(12), 2625-2626.

• Klein, A., Tate, D. J., Jayasekeran, V., Hourigan, L., Singh, R., Brown, G., ... & Sidhu, M. (2019). Thermal ablation of mucosal defect margins reduces adenoma recurrence after colonic endoscopic mucosal resection. Gastroenterology, 156(3), 604-613

• Klein, A., & Bourke, M. J. (2015). Advanced polypectomy and resection techniques. Gastrointestinal Endoscopy Clinics, 25(2), 303-333.

• Burgess, N. G., Bassan, M. S., McLeod, D., Williams, S. J., Byth, K., & Bourke, M. J. (2017). Deep mural injury and perforation after colonic endoscopic mucosal resection: a new classification and analysis of risk factors. Gut, 66(10), 1779-1789.

• Tate, D. J., Bahin, F. F., Desomer, L., Sidhu, M., Gupta, V., & Bourke, M. J. (2018). Cold-forceps avulsion with adjuvant snare-tip soft coagulation (CAST) is an effective and safe strategy for the management of non-lifting large laterally spreading colonic lesions. Endoscopy, 41(01), 52-62.

• Bahin, F. F., Rasouli, K. N., Byth, K., Hourigan, L. F., Singh, R., Brown, G. J., ... & Bourke, M. J. (2016). Prediction of clinically significant bleeding following wide-field endoscopic resection of large sessile and laterally spreading colorectal lesions: a clinical risk score. American Journal of Gastroenterology, 111(8), 1115-1122.

• Albéniz, E., Fraile, M., Ibáñez, B., Alonso-Aguirre, P., Martínez-Ares, D., Soto, S., ... & Múgica, F. (2016). A scoring system to determine risk of delayed bleeding after endoscopic mucosal resection of large colorectal lesions. Clinical Gastroenterology and Hepatology, 14(8), 1140-1147.

• Bahin, F. F., Rasouli, K. N., Williams, S. J., Lee, E. Y., & Bourke, M. J. (2016). Prophylactic clipping for the prevention of bleeding following wide-field endoscopic mucosal resection of laterally spreading colorectal lesions: an economic modeling study. Endoscopy, 48(08), 754-761
