# Supplementary material for: Training in endoscopic mucosal resection: effectiveness and clinical utility of a short course for practicing endoscopists
Source: J Can Assoc Gastroenterol. 2025 Jun 21;8(5):184–91. doi: 10.1093/jcag/gwaf015 (PMC12551745; doi:10.1093/jcag/gwaf015)
Supplement: gwaf015_Supplementary_Data [file gwaf015_supplementary_data.zip › DoCTRINE-Guideline-Checklist - AK_jan 20.docx]

**Defined Criteria To Report INnovations in Education (DoCTRINE)**

*Citation:* Blanco M, Prunuske J, DiCorcia M, Learman LA, Mutcheson B, Huang GC. The DoCTRINE Guidelines: Defined Criteria To Report INnovations in Education. Academic Medicine. 2022 May 1;97(5):689-695.

| **Introduction** | **Y/N** |
| --- | --- |
| 1. Need for the curriculum | Y |
| 1. Review of relevant literature, theories, models, or published curricula | Y |
| 1. Unique contribution of the curriculum to the literature | Y |
| **Curriculum Development** | **Y/N** |
| 1. Purpose/goals of the curriculum | Y |
| 1. Outcome-based learning objectives | Y |
| 1. Target population of learners | Y |
| **Curriculum implementation** | **Y/N** |
| 1. Instructional setting for curriculum delivery | Y |
| 1. Resources for implementing the curriculum | Y |
| 1. Description of instructional methods | Y |
| 1. Methods to evaluate achievement of outcome-based learning objectives | Y |
| 1. Origin of evaluation instrument(s) | Y |
| **Results** | **Y/N** |
| 1. Number of learners participating in the curriculum | Y |
| 1. Number of participants included in the evaluation | Y |
| 1. Evidence of achievement of outcome-based learning objectives | Y |
| **Discussion** | **Y/N** |
| 1. Summary of findings | Y |
| 1. Interpretation of findings in relation to the existing literature | Y |
| 1. Lessons learned from the implementation of the curriculum | Y |
| 1. Limitations of the evaluation of the curriculum | Y |
| 1. Describes future implications of the curriculum | Y |

**Training in Endoscopic Mucosal Resection: Effectiveness and Clinical Utility of a Short Course for Practicing Endoscopists**

Ahmed Kayal MD^1,2,7^, Sylvain Coderre MD MSc^1,2^, Maitreyi Raman MD MSc^1,2^, Heather L Hill^3^, Stephanie Jaunin^3^, Diana Kerrison RN^4^, Adrian Harvey MD MSc MEd^6^, Kevin McLaughlin MB ChB PHD^2,5^, Steven J. Heitman MD MSc^1,2,4^

1. Department of Medicine, Division of Gastroenterology and Hepatology, University of Calgary, Alberta, Canada
2. Department of Community Health Sciences, University of Calgary, Alberta, Canada
3. Advanced Technical Skills Simulation Laboratory, Cumming School of Medicine, University of Calgary, Alberta, Canada
4. Forzani & MacPhail Colon Cancer Screening Centre, Alberta Health Services (Calgary Zone), Alberta, Canada
5. Department of Medicine, Division of Nephrology, University of Calgary, Alberta, Canada
6. Departments of Surgery and Oncology, University of Calgary, Alberta, Canada
7. Department of Medicine, Rabigh Branch. King Abdulaziz University, Jeddah, Saudi Arabia

**Corresponding Author:**

Ahmed Kayal, MD, MSc

Assistant Professor

Department of Medicine – Rabigh Branch

King Abdulaziz University. Jeddah. Saudi Arabia

Tel: +966556626562

E-mail: atkayal@kau.edu.sa
